# Aversion, interpretation and determinability: Three factors of uncertainty that may play a role in psychopathology

**DOI:** 10.3758/s13415-023-01068-6

**Published:** 2023-02-15

**Authors:** Caroline Moul, Hilary J. Don, Evan J. Livesey

**Affiliations:** 1 School of Psychology, University of Sydney, Sydney, NSW 2006 Australia; 2 Division of Psychology & Language Sciences, University College London, London, UK

**Keywords:** Tolerance of uncertainty, Individual differences, Anxiety, Associative learning, Learning window width

## Abstract

This opinion piece considers the construct of tolerance of uncertainty and suggests that it should be viewed in the context of three psychological factors: uncertainty aversion, uncertainty interpretation, and uncertainty determinability. Uncertainty aversion refers to a dislike of situations in which the outcomes are not deterministic and is similar to conventional conceptions of (in)tolerance of uncertainty. Uncertainty interpretation refers to the extent to which variability in an observed outcome is interpreted as random fluctuation around a relatively stable base-rate versus frequent and rapid changes in the base-rate. Uncertainty determinability refers to the (actual or perceived) capacity of the individual to generate *any* meaningful expectancy of the uncertain outcome, which may be undeterminable if predictions are updated too quickly. We argue that uncertainty interpretation and determinability are psychological responses to the experience of probabilistic events that vary among individuals and can moderate negative affect experienced in response to uncertainty. We describe how individual differences in basic parameters of associative learning (modelled by a simple learning window) could lead to this variation. To explain these hypotheses, we utilise the distinction between aleatory uncertainty (the inherent unpredictability of individual stochastic events) and epistemic uncertainty (obtainable knowledge that the individual lacks or perceives to be lacking). We argue that when expectancies are updated quickly, epistemic uncertainty will dominate the individual’s representation of the events around them, leading to a subjective experience of the world as one that is volatile and unpredictable.

Many of our behavioural decisions are based on prior experience. If a behaviour has generally resulted in a positive outcome, then we are likely to engage in that behaviour again. If a behaviour resulted in predominantly negative outcomes, we are likely to avoid repeating it in the future. The nature of behaviour-outcome associations often is stochastic: outcomes occur probabilistically about a stable base rate. For example, you might have a temperamental cat who purrs on average 7 of every 10 times you stroke it, but scratches you on the other occasions. An individual who decides not to stroke the cat, given this 3:7 odds ratio of punishment to reward, may be described as risk averse compared with an individual who decides to stroke the cat again given the same experiences. One explanation for risk aversion in this context could be a heightened sensitivity to negative outcomes: “punishment sensitivity.” Indeed, this is the conventional view that has been adopted in research on anxiety (Aylward et al., 2019; Torrubia et al., 2001), and the inverse relationship (low levels of risk aversion associated with punishment insensitivity) has been argued to be important for other psychopathologies (Mitchell et al., 2002; Moul et al., 2012). However, there are mechanisms that can generate the same predicted risk aversion without having to assume individual differences in sensitivity to reward or punishment. We will describe three potential mechanisms for individual differences in risk aversion in the context of probabilistic outcomes.

As this special issue clearly indicates, tolerance for uncertainty has been recognised as an important transdiagnostic construct across anxiety disorders and related psychopathologies (Carleton et al., 2012; Gu et al., 2020) and has been argued to have a genetically derived, biological basis (Brosschot et al., 2016; Hirsh et al., 2012). The exact definition of “tolerance of uncertainty” varies, but the main tenet is that people differ in how they respond emotionally and behaviourally to events with a probabilistic outcome, that is, a situation in which an outcome sometimes (but not always) follows from the known antecedent cue that predicts its occurrence.

## Defining uncertainty

The concept of uncertainty itself takes different forms across psychology and decision making research (see Kozyreva and Hertwig (2021) for a discussion of different taxonomies of uncertainty). There is a longstanding distinction drawn by Knight (1921) between risk and uncertainty. Whereas risk refers to known or at least *knowable* information about the long-run probability of an event, Knightian uncertainty refers to (a lack of) information that cannot pragmatically be measured—perhaps because it pertains to a unique event for which there is no prior knowledge, or because prior measures are likely to be obsolete (causal states that determine the probabilities have changed)—and for which subjective estimates must be relied upon. This distinction has shaped economics, psychology, and related disciplines over the past hundred years.

## Aleatory uncertainty and epistemic uncertainty

A related distinction, which is particularly useful for our arguments, draws a psychological boundary between aleatory uncertainty and epistemic uncertainty (Fox & Ülkümen, 2011; Hacking, 2006). Aleatory uncertainty refers to the unpredictability of individual events, by virtue of the stochasticity of probabilistic distributions. This aleatory uncertainty is broadly aligned with the Knightian concept of risk in that it concerns information that is available given sufficient sampling or experience—an estimation of the base rate can be calculated despite probabilistic variability.

Epistemic uncertainty refers more directly to the *ignorance* of the individual about the state of the events in question. That is, it refers to the state of not yet knowing the discoverable properties of the events. Uncertainty, therefore, is widely recognised to take different forms, and the information that an observer possesses (e.g., about the probability of the relevant events) and uses (e.g., to make predictions about the future) is critical to these forms.

The literature on aleatory versus epistemic uncertainty highlights the fact that uncertainty must be considered in the context of what the individual has learned. Assuming that this knowledge might well be different across individuals, it leads to the possibility that uncertainty might not be experienced in the same way by learners given the exact same probabilistic sequence of events. We outline several psychological factors that might contribute to individual differences in tolerance of uncertainty, based on variations in the way information is accumulated and used to form judgments and predictions.

## Uncertainty aversion

Individuals with low tolerance of uncertainty may have negative emotional responses to uncertainty and may avoid behaviours for which the outcome is uncertain (Grupe & Nitschke, 2013; Gu et al., 2020). Those with a low tolerance may determine the 3:7 odds of being scratched by the cat to be unacceptable, because they are averse to not being sure of the outcome beyond knowing that there is a 30% chance of being scratched on any given occasion. The implication here is that the individual is aware of the underlying probabilities, that is, of estimable risk. Such a view assumes that the individual has reasonable knowledge of the objective probability of the outcome (Dewitt et al., 2020) *and* that this probability is sufficiently stable to inform future choice: they appreciate the aleatory uncertainty involved, and their decision to approach or avoid the cat is a response to calculated risk. According to this account, tolerance of uncertainty is driven by the extent to which the individual feels averse to the odds of an unfavourable outcome and the extent to which they accept this risk. For our purposes, and in line with previous literature, we are simply defining *uncertainty aversion* as the extent to which an individual experiences negative thoughts or sensations in response to a situation for which the outcome is not deterministic. In this regard, the majority of experimental research in intolerance of uncertainty could be described as assessing uncertainty aversion by comparing emotional, physiological, and behavioural responses to nondeterministic versus deterministic cues (Lin et al., 2014; Morriss et al., 2019). Thus, in isolation, this *uncertainty aversion* account is not concerned with the extent to which an individual experiences stability in the odds over which risk is defined or whether they have been successful in forming an estimation of the odds in the first place. However, we argue that the subjective stability of the odds and the individual’s ability to form an estimation of the odds, influence their experience of probabilistic environments as possessing aleatory versus epistemic uncertainty. It is these two factors that we address next.

## Estimating the odds

Whereas uncertainty aversion describes a dislike of situations in which the outcomes are not deterministic, a consideration of the mechanisms underlying the learning processes that give rise to contingency information suggest other factors may also be at play. There are numerous models of learning and of making choices under uncertainty, many inspired by simple prediction error algorithms of associative learning and reward learning (Behrens et al., 2007). Differences in the values taken by parameters of these algorithms, such as learning rate and decay rate, result in different amounts of information being used to form expectancies of outcomes based on prior experience (Behrens et al., 2007; Dayan et al., 2000). Most models of this nature make a fundamental prediction about the relationship between the speed of information updating and the manner in which past events control our current predictions. When the updating of our predictions is fast (e.g., achieved through a high learning rate), our expectations about future events are dominated by what has happened on the occasions that we most recently experienced: a small window of recent prior experiences control our expectations and behaviours. When updating is slower, that window of prior events that have a functional effect on our current behaviour stretches further back in our leaning history (Moul et al., 2021) .

**Fig. 1:**
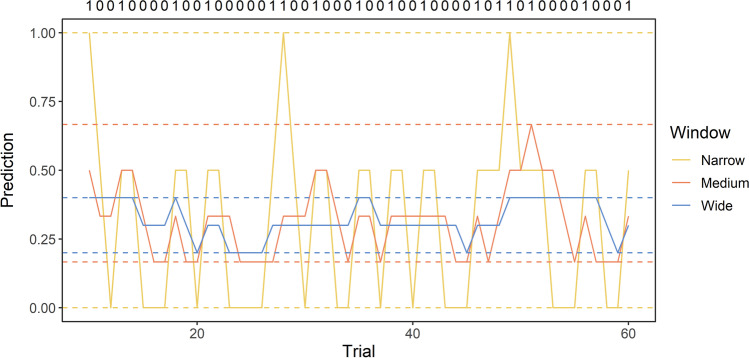
Expected outcome predictions for a trial sequence with a 3:7 outcome base-rate for individuals with a narrow, medium, and wide window width. Solid lines indicate outcome predictions on each trial. Dashed lines indicate the range of predictions for each window width. Wider window widths will change value less frequently, whereas narrower window widths will produce more frequent changes and less stability over time

We have previously demonstrated that this property can be modelled simply and effectively by a learning window (Moul et al., 2021): the next expected outcome is generated from the simple average of the outcomes that fall within a window of experiences that project backwards over prior history starting from the most recent outcome. Given a probabilistic behaviour-outcome association with stochastic variation about a stable base rate, a normal distribution of individual differences in the width of this window results in different expected outcomes. Akin to the effects of fast learning and fast decay rates, a narrow window generates outcome estimates based on only the average of the most recent outcome events. Wider windows form outcome estimates based on the average of a longer history of outcomes. As illustrated in Fig. 1, the result of this is that individuals with a narrower window generate expectancies that fluctuate more wildly from the objective base-rate than individuals with a wider window (Moul et al., 2021).

This computational approach to modelling prediction generation is incredibly simple, and not surprisingly, it is formally very similar to several others reported in research on decision making from experience and reinforcement learning. It could be viewed as an extension of the *natural mean heuristic* (Hertwig & Pleskac, 2008; Sutton & Barto, 2018), where a mean expected value is calculated from instances experienced in the past, but the sampling of past instances to derive this mean are constrained in a consistent way (i.e., a limited number of recent events). Other models use similar constraining assumptions. For instance, Ashby and Rakow (2014) used a sliding window of information model that calculates an expected value from a window of the most recent experiences and showed that, for modelling subjective valuations from experience, the model compared favourably to alternatives that used all experiences to estimate expected values. Erev et al.’s (2008) k-sampler model works off a similar principle of deriving a prediction based on the mean value calculated over a limited set of past instances, although the sample of instances used to make valuations is chosen less deterministically. As noted, the width of this window determines properties of information use and information loss that are similar to other computational approaches such as learning updated by a delta rule (Rescorla & Wagner, 1972). The point of significance here is that we hypothesise that the width of this window—the extent of past instances that have a functional influence on an individual’s predictions—may vary in a trait-like way across individuals. Some may be disposed to using very few (and only very recent) events, whereas others may form more long-run predictions based on a wider window of events. Importantly, if this is true, then it may have consequences for the way people experience, interpret, and navigate nondeterministic events.

## Outcome estimates as a function of learning window width

The fluctuations in expected outcome produced by a narrower window would result in a diminished ability to determine the stable base-rate of the outcome. The implications of this are twofold. 1) The individual may interpret the underlying base-rate as volatile; rather than a single stable base-rate, the individual may interpret their experiences as being indicative of base-rates that change relatively frequently (Gershman et al., 2010). In this situation, even if the individual is able to estimate outcome probabilities based on what has recently happened, any prediction they make must be considered alongside the belief that the base-rate may change at any moment, rendering their past learning largely irrelevant to their estimate of the base-rate in the future. This would be a key basis for perceiving high epistemic uncertainty even in the face of considerable past experience. In this situation, the relevance of past experience is called into question resulting in the perception of insufficient information. 2) The individual may be unable to determine *any* useful estimate of the current base-rate. If the probability of the outcome appears to be changing quickly then the learner may not be able to form an estimate based on anything more than the outcome they have just observed. Further, they may internalise this failing as a property of themselves rather than the stochastic environment that surrounds them. The *interpretation* of an action-outcome contingency as one possessing a volatile base-rate and the belief that the base-rate cannot be *determined* constitute two examples where knowledge representations may emerge differently from simple learning mechanisms.

## Interpretation and determinability

To be clear, we are defining *uncertainty interpretation* as the degree to which predictions and judgments generated from past learning are viewed as stable and reliable (i.e., reflecting the experience of slow-changing base-rate probabilities derived from relatively constant underlying causes) or volatile and unreliable (i.e., reflecting the experience of rapidly changing base-rates derived from highly variable underlying causes). As learning window width decreases, the outcome predictions that are generated fluctuate more wildly around the actual base rate such that the perception of volatility, and of variable underlying causes, is increased. *Uncertainty determinability* is defined here as the extent to which *any* estimate of the next expected outcome is able to be generated and, in turn, the learner’s representation of their capacity to generate such a prediction.

As window width diminishes, the individual’s capacity to respond quickly to change is enhanced, but this comes at the cost of poorer precision in estimating base-rates that are stable in the longer term. Estimates based on narrower windows fluctuate more with incidental variation in probabilistic outcomes and necessarily convey less information about the base rate. A corollary of these limitations is that a stable probabilistic action-outcome contingency could be experienced as unstable and/or incalculable, and thus not possessing a base-rate that can be determined. Without an estimation of the stable base-rate of an outcome, the individual experiences more epistemic uncertainty where another individual might experience aleatory uncertainty associated with a quantifiable risk. If the degree of risk is unknown, the individual is left with no situation-specific information to guide their behavioural choices and, at best, they have to extrapolate from other sources (i.e., make an educated guess). At worst, the individual may hold the belief that they are helpless and left with no choice but to make completely uninformed decisions.

The perceived absence of salient or sufficient information to guide behaviour, and the aversion to this also features in current definitions of intolerance of uncertainty (Carleton, 2016). As such, the definitions we present are not designed to replace current conceptualisations of intolerance of uncertainty. Rather, we propose them as potential mechanisms that could explain individual differences in intolerance of uncertainty. Critically, we argue that individual differences in basic parameters of associative learning can lead to variation in the ability to generate outcome predictions from probabilistic events, which influences perceptions of knowledge state.

The manner in which individuals use past experiences to generate expectancies and predictions (i.e., guided by a relatively wide vs. narrow window of past events) may have downstream implications for how uncertainty around a probabilistic outcome is interpreted and how and whether long-run event probabilities can be intuitively calculated by the individual. Why should that matter for an individual’s intolerance of uncertainty? People tend to show an aversion to choices that have uncertain outcomes when they believe that the source of the uncertainty is their own lack of knowledge about something that could otherwise be known (Fox & Tversky, 1995; Heath & Tversky, 1991). This broadly corresponds to the idea that epistemic uncertainty, in particular, is aversive. Alternatively, when the uncertainty is due to factors that the individual believes are unknowable—the inherent randomness of flipping a coin, for instance—then the (aleatory) uncertainty does not elicit negative affect in the same way.

Learning window width influences the *interpretation* and *determinability* of uncertainty. It may not be the case that individuals who decide not to stroke the cat again find the 3:7 odds of being scratched unacceptable (uncertainty aversion) and may not find the scratch itself particularly aversive. Rather, it may be that they have experienced the odds of being scratched as constantly changing (uncertainty interpretation) or that they have been unable to determine any stable odds of being scratched (uncertainty determinability). In either event, the individual is left with no stable information on which to base their decision. Objectively, their experience of the cat is the same as someone with a wide window; subjectively their experience is less stable, less predictable, and less useful.

Understanding the relative contributions of these three mechanisms to the tolerance of uncertainty is important given individual differences in uncertainty tolerance, its association with psychopathologies (Carleton et al., 2012; Gu et al., 2020), and the research literature that implicates aberrant associative learning mechanisms in anxiety disorders (Grupe & Nitschke, 2013). Future research needs to develop ways to explicitly test these three components. One approach may be to give learners different levels of scaffolding in how they represent a probabilistic task in order to regulate how uncertainty is interpreted. For example, task instructions that include explicit base-rate expectations should limit interpretation of differences in intolerance of uncertainty to those of uncertainty aversion alone. Indeed, research has begun to look at the impact of contingency instructions on the relationship between intolerance of uncertainty and indices of threat conditioning and extinction (Morriss et al., 2022; Morriss & van Reekum, 2019). Alternatively, results from tasks that index the speed of updating and outcome expectancies independently from risk and decision making could be used to gauge whether these factors have a relationship to anxiety and other indicators of risk of psychopathology.

When we conduct research in the area of learning and decision-making, we tend to make the assumption that the contingencies we program are the contingencies experienced by our participants. Thus, we expect our participants to behave in ways that accord with those contingencies. However, individual differences in basic learning parameters, or the width of the functional window of past events being used to make predictions, effectively change participants’ experiences and representations of those contingencies. One participant’s emotions and behaviours may be appropriate to the experimental world that they inhabit whilst being radically different from another participant’s, which are equally as appropriate to the experimental world that they experience. We often interpret these differences in behaviour in terms of personality or psychopathology: risky behaviour, anxiety, poor decision-making. Recently, it has been argued that we need to take more time to consider how individuals represent task structure in probabilistic decision-making tasks (Szollosi et al., 2022). In line with this argument, when it comes to understanding psychopathology, we may need to consider whether individual differences in simple parameters of associative learning, such as those that can be modelled by learning window width, may affect people’s representations of uncertain events, and how these representations can give rise to dramatic variations in our experiences of, beliefs about, and reactions to events in our world.

## References

[CR1] Ashby NJ, Rakow T (2014). Forgetting the past: Individual differences in recency in subjective valuations from experience. Journal of Experimental Psychology. Learning, Memory, and Cognition.

[CR2] Aylward J, Valton V, Ahn W-Y, Bond RL, Dayan P, Roiser JP, Robinson OJ (2019). Altered learning under uncertainty in unmedicated mood and anxiety disorders. Nature Human Behaviour.

[CR3] Behrens TEJ, Woolrich MW, Walton ME, Rushworth MFS (2007). Learning the value of information in an uncertain world. Nature Neuroscience.

[CR4] Brosschot JF, Verkuil B, Thayer JF (2016). The default response to uncertainty and the importance of perceived safety in anxiety and stress: An evolution-theoretical perspective. Journal of Anxiety Disorders.

[CR5] Carleton RN (2016). Into the unknown: A review and synthesis of contemporary models involving uncertainty. Journal of Anxiety Disorders.

[CR6] Carleton RN, Mulvogue MK, Thibodeau MA, McCabe RE, Antony MM, Asmundson GJG (2012). Increasingly certain about uncertainty: Intolerance of uncertainty across anxiety and depression. Journal of Anxiety Disorders.

[CR7] Dayan P, Kakade S, Montague PR (2000). Learning and selective attention. Nature Neuroscience.

[CR8] Dewitt S, Fenton NE, Liefgreen A, Lagnado DA (2020). Propensities and second order uncertainty: A modified taxi cab problem. Frontiers in Psychology.

[CR9] Erev I, Glozman I, Hertwig R (2008). What impacts the impact of rare events. Journal of Risk and Uncertainty.

[CR10] Fox C, Tversky A (1995). Ambiguity aversion and comparative ignorance*. The Quarterly Journal of Economics.

[CR11] Fox C, Ülkümen G, Brun W, Kirkebøen G, Montgomery H (2011). Distinguishing two dimensions of uncertainty. * Perspectives on Judgment and Decision Making *.

[CR12] Gershman SJ, Blei DM, Niv Y (2010). Context, learning, and extinction. Psychological Review.

[CR13] Grupe DW, Nitschke JB (2013). Uncertainty and anticipation in anxiety: An integrated neurobiological and psychological perspective. Nature Reviews Neuroscience.

[CR14] Gu Y, Gu S, Lei Y, Li H (2020). From uncertainty to anxiety: How uncertainty fuels anxiety in a process mediated by intolerance of uncertainty. Neural Plasticity.

[CR15] Hacking I (2006). * The Emergence of Probability: A Philosophical Study of Early Ideas about Probability, Induction and Statistical Inference *.

[CR16] Heath C, Tversky A (1991). Preference and belief: Ambiguity and competence in choice under uncertainty. Journal of Risk and Uncertainty.

[CR17] Hertwig R, Pleskac TJ, Chater N, Oaksford M (2008). The game of life: How small samples render choice simpler. * The Probabilistic Mind:Prospects for Bayesian Cognitive Science *.

[CR18] Hirsh JB, Mar RA, Peterson JB (2012). Psychological entropy: A framework for understanding uncertainty-related anxiety. Psychological Review.

[CR19] Knight F (1921). *Risk, Uncertainty, and Profit*.

[CR20] Kozyreva A, Hertwig R (2021). The interpretation of uncertainty in ecological rationality. Synthese.

[CR21] Lin, H., Gao, H., You, J., Liang, J., Ma, J., Yang, N., ... Jin, H. (2014). Larger N2 and smaller early contingent negative variation during the processing of uncertainty about future emotional events. *International Journal of Psychophysiology, 94*(3), 292-297. 10.1016/j.ijpsycho.2014.10.004 10.1016/j.ijpsycho.2014.10.004 25312204

[CR22] Mitchell DGV, Colledge E, Leonard A, Blair RJR (2002). Risky decisions and response reversal: Is there evidence of orbitofrontal cortex dysfunction in psychopathic individuals?. Neuropsychologia.

[CR23] Morriss J, van Reekum CM (2019). I feel safe when i know: Contingency instruction promotes threat extinction in high intolerance of uncertainty individuals. Behaviour Research and Therapy.

[CR24] Morriss J, Saldarini F, van Reekum CM (2019). The role of threat level and intolerance of uncertainty in extinction. International Journal of Psychophysiology.

[CR25] Morriss J, Bradford DE, Wake S, Biagi N, Tanovic E, Kaye JT, Joormann J (2022). Intolerance of uncertainty and physiological responses during instructed uncertain threat: A multi-lab investigation. Biological Psychology.

[CR26] Moul C, Killcross S, Dadds MR (2012). A model of differential amygdala activation in psychopathy. Psychological Review.

[CR27] Moul C, Robinson OJ, Livesey EJ (2021). Antisocial learning: Using learning window width to model callous-unemotional traits?. Computational Psychiatry.

[CR28] Rescorla R, Wagner A (1972). A theory of Pavlovian conditioning: The effectiveness of reinforcement and non-reinforcement Classical Conditioning: Current Research and Theory.

[CR29] Sutton RS, Barto AG (2018). * Reinforcement learning: An introduction *.

[CR30] Szollosi, A., Donkin, C., & Newell, B. R. (2022). Toward nonprobabilistic explanations of learning and decision-making. *Psychological Review*. 10.1037/rev0000355 10.1037/rev0000355 35389718

[CR31] Torrubia R, Ávila C, Moltó J, Caseras X (2001). The sensitivity to punishment and sensitivity to reward questionnaire (SPSRQ) as a measure of Gray's anxiety and impulsivity dimensions. Personality and Individual Differences.

